# Growth overshoot and seasonal size changes in the skulls of two weasel species

**DOI:** 10.1098/rsos.160947

**Published:** 2017-01-25

**Authors:** Scott LaPoint, Lara Keicher, Martin Wikelski, Karol Zub, Dina K. N. Dechmann

**Affiliations:** 1 Department of Migration and Immuno-Ecology, Max Planck Institute for Ornithology, Am Obstberg 1, Radolfzell 78315, Germany; 2 Department of Biology, University of Konstanz, Universitätsstrasse 10, Konstanz 78457, Germany; 3 Mammal Research Institute, Waszkiewicza 1, Białowieża 17-230, Poland

**Keywords:** body size, Dehnel's phenomenon, morphology, *Mustela*, ontogeny, sexual dimorphism

## Abstract

Ontogenetic changes in mammalian skulls are complex. For a very few species (i.e. some *Sorex* shrews), these also include seasonally driven, bidirectional size changes within individuals, presumably to reduce energy requirements during low resource availabilities. These patterns are poorly understood, but are likely most pronounced in high-metabolic species with limited means for energy conservation. We used generalized additive models to quantify the effect of location, Julian day, age and sex on the length and depth of 512 and 847 skulls of stoat (*Mustela erminea*) and weasel (*M. nivalis*) specimens collected throughout the northern hemisphere. Skull length of both species varies between sexes and geographically, with stoat skull length positively correlated with latitude. Both species demonstrate seasonal and ontogenetic patterns, including a rare, absolute growth overshoot in juvenile braincase depth. Standardized braincase depths of both species peak in their first summer, then decrease in their first winter, followed by a remarkable regrowth that peaks again during their second summer. This seasonal pattern varies in magnitude and timing between geographical regions and the sexes, matching predictions of Dehnel's phenomenon. This suggests implications for the evolution of over-wintering strategies in mammals, justifying further research on their mechanisms and value, with implications for applied osteology research.

## 1. Introduction

Seasonal fluctuations in resources are a common but, from an evolutionary viewpoint, difficult problem for animals to solve. Species respond to such extrinsic challenges with behavioural adaptations, such as food caching [1], or physiologically, for example by suppressing bone resorption during hibernation [2]. These strategies are believed to facilitate the efficient use of energy and ultimately increase survival. Species also exhibit population-level morphological adaptations, for example shifts in mammal body size—arguably the most important trait of a species and individual [3,4]—driven by increased global temperatures [5–8]. Biologists have long been intrigued by body size (e.g. skull length), quantifying its variation and identifying its drivers, yielding descriptions of ecological patterns and ‘rules’ (e.g. Bergmann's or Allen's rules). These patterns probably arise in response to several variables combined (i.e. resource availability), not solitary factors (e.g. latitude or precipitation) (*sensu* [9]).

In addition to these extrinsic influences, individuals also experience a lifetime of intrinsic ontogenetic challenges [3,10–12]. For example, ontogenesis for mammalian carnivores includes changes in skull shape and size as they mature from altricial juveniles to bone-crushing adults [13]. Changes in the shape and relative size of the skull of growing individuals are common in mammals [3,10], whereas absolute skull size principally increases until they are fully grown, except for some bone resorption in very old individuals [14]. This stability in adult skull shape and size is governed by the ossification of the skull, including the fusing of sutures and the formation of osteoclasts [15,16].

Efforts to quantify potential extrinsic drivers of skull size must therefore also account for the ontogenetic patterns described above, particularly for species whose life-history stage transitions coincide with intra-annual environmental patterns such as seasonal fluctuations in resource availability. In contrast with more common over-wintering coping strategies, e.g. torpor or migration, a few species have evolved an alternative strategy: reduce their absolute energy requirements during harsh conditions [17]. Dehnel's phenomenon, first documented in red-toothed shrews (Soricinae), describes the seasonal and reversible size change of the skull [17,18], skeleton [19], brain [20] and several major organs [21] of an individual. Like others before him [22,23], Dehnel recognized remarkable ontogenetic changes in *Sorex* skulls where juvenile skulls demonstrate a growth overshoot in both skull length and depth. Their skull then decreases in both dimensions from their first summer until they reach a minimum midway through their first winter, and later regrow again as they reach adulthood at the end of their first winter reaching a second size peak as adults by their second summer [17]. The juvenile absolute growth overshoot is rare in vertebrates; only known for these *Sorex* shrews and for the brains of captive mink (*M. vison*; [24]) and ferret (*M. putorius furo*; [25]), and the skulls of weasel (*M. nivalis*; [26]) and polecat (*M. putorius*; [27]). Importantly, Dehnel hypothesized that these changes were seasonally driven and in addition to ontogenetic effects. His hypothesis has since been supported with additional work on *Sorex* species, focusing especially on the brain and skull.

The seasonality of the phenomenon is accepted and there is some evidence for its adaptive advantage, yet there is limited consensus on the specific drivers [28]. The benefit of being relatively small during resource availability lulls is well documented at the population level for several species, where relatively small individuals are more likely to survive [29–32]. At the individual level, proactively reducing energy expenditure or requirements, for example through food caching or torpor, is a successful strategy for coping with reduced resource availability [33], but is not feasible for species with very high metabolisms and year-round activity patterns (e.g. shrews [34]). Instead, shrews are able to reduce their body mass, including several energy-expensive organs and the brain, reducing their absolute energy requirements when resource availability is low (*sensu* [35,36]).

To better understand the evolution of the Dehnel's phenomenon, it is not only important to identify other species where individuals exhibit seasonal size changes, but also to distinguish between extrinsic and intrinsic drivers of these size changes. Stoats (*M. erminea*) and weasels (*M. nivalis*) share many ecological, physiological and behavioural traits with *Sorex* shrews, including limited abilities for reducing heat loss [37], high metabolisms [37], predatory behaviour [38] and the inability to enter torpor or hibernate [37]. Additionally, the reproductive strategies of these weasel species differ and offer opportunities for testing hypotheses on energy limitation. Males of both species provide no parental care for offspring, enabling them to invest all available energies into themselves, and should therefore exhibit the greatest seasonal size patterns. Female stoats produce a single litter per year, but not until their second year, and therefore should exhibit a seasonal pattern similar to males, but to a lesser degree. Female weasels however, can produce two litters per year and mate within a few weeks of their birth, and are therefore unlikely to exhibit a seasonal size pattern as adults owing to them diverting more energies to their offspring [38]. Although previous work has documented unusual juvenile growth overshoots in related species [24,25,27] including weasels [26], no studies have done so for stoats, nor investigated seasonal patterns in stoat or weasel skulls. Our own preliminary work suggests that weasel specimens collected from northeastern Poland exhibit Dehnel's phenomenon-like seasonal patterns in braincase depth (BD), but further work was recommended to distinguish between the confounding effects of intrinsic and extrinsic variables [39].

We recorded condylobasal length (CBL), braincase breadth (BB) and braincase depth (BD), commonly used metrics in the study of Dehnel's phenomenon, on the skulls of stoat and weasel specimens collected from across the northern hemisphere during the last 120 years (Dryad: http://dx.doi.org/10.5061/dryad.g57g1 [40]). We standardized BB and BD with CBL (BB_s_, BD_s_) to account for known population and species-level effects, i.e. latitudinal, sex and inter-annual variability in skull size [41]. We used generalized additive models (GAMs; [42]) to identify age-, latitude-, season- and sex-based patterns in BD_s_. Because of their similarities with *Sorex* shrews, we hypothesized that these species would likewise demonstrate seasonal patterns in BD. We predicted that (i) both species would demonstrate juvenile growth overshoots in absolute skull depth similar to those exhibited by other *Mustela* species, (ii) the presence and magnitude of seasonal patterns would be positively related to latitude, and (iii) that males of both species would exhibit greater plasticity in skull depth. Our globally sourced dataset and powerful statistical approach allowed us to systematically account for and distinguish between the drivers for known (i.e. age, sex dimorphism and latitudinal variation) and potentially hitherto unknown seasonal skull size variation in these carnivores.

## 2. Material and methods

### 2.1. Study species

Stoats and weasels (Mustelidae) inhabit many of the grasslands, forests and mountainous ecosystems of the Holarctic, ranging between 30 and 80° N latitude [38]. They are strict carnivores, with long, narrow body shapes and are sexually dimorphic in size [38]. Both species exhibit substantial variation in body length and mass across their range, even within relatively small geographical regions (e.g. Poland [26,43]): female weasel (excluding *M. n. subpalmata*) body lengths of 136–183 mm and masses of 31–82 g, male weasel body lengths of 151–222 mm and masses of 53–193 g, female stoat body lengths of 153–264 mm and masses of 45–213 g and male stoat body lengths of 166–297 mm and masses of 59–334 g [38]. Both species are relatively short-lived in natural populations: female and male stoats live up to 4 years, while female and male weasels live up to 3 years, but most for only 1 year [38]. Both species are active throughout the year and day, with alternating bouts of resting and hunting to fuel their extremely high metabolisms [37,38]. Their body shapes, rather poor abilities for reducing heat loss [37], and high metabolisms make thermoregulation very challenging for both species, especially those in northern latitudes.

### 2.2. Data collection

Linear measurements were taken on the intact skulls of weasel and stoat specimens housed within the following natural history collections, listed by sample sizes of weasels and stoats, respectively: Muséum d‘Histoire Naturelle Brussels (454, 184), Mammal Research Institute Białowieża (502, 0), National Museum of Natural History Washington (143, 200), Royal Ontario Museum Toronto (0, 184), Finnish Museum of Natural History Helsinki (106, 74), Museum of Vertebrate Zoology Berkeley (60, 120), University of Michigan Museum of Zoology (93, 52), New York State Museum Albany (1, 68), Muséum d‘Histoire Naturelle Geneva (35, 33), Michigan State University Museum (34, 24), Cornell University (1, 30), Museum für Naturkunde Berlin (25, 6), North Carolina Museum of Natural Sciences Raleigh (10, 9), and the North Carolina State University (2, 4). The species, sex and the date and location of collection were recorded for each specimen. Care was taken to distinguish between dates of preparation and dates of collection, at times requiring verification through original ledgers and collection manager receipts of samples from expeditions. Specimens with uncertain dates were not included in our analyses. To investigate geographical patterns in species skull sizes, we georeferenced specimens (precision ±0.1 decimal degrees) using their locality information when latitudinal and longitudinal information was not available, otherwise we used the provided coordinates. Individuals were assigned to one of three age classes (juvenile, sub-adult or adult) following guidelines proposed by King [44] and Schmidt [26] (electronic supplementary material, figure S1). Note that our naming convention for these age categories should not imply stages of maturation, but we probably classified independent individuals aged less than four months as ‘juveniles’, individuals generally aged between four and seven months with mostly ossified skulls but without pronounced nuchal or sagittal crests as ‘sub-adults’, and individuals older than seven months with fully ossified skulls and at least partially developed nuchal crests as ‘adults’.

We recorded the CBL (measured from the posterior of the occipital condyles to the anterior tip of the premaxilla), breadth of braincase (BB; measured at the greatest width of the braincase posterior to the zygomatic arches) and the BD (measured perpendicularly from the basioccipital, i.e. omitting the auditory bullae, to the top of the braincase, avoiding the sagittal crest when present) using digital calipers (±0.01 mm precision). In addition to these linear metrics, we also estimated a proxy for braincase volume for weasel (*n* = 164) and stoat (*n* = 77) specimens by calculating the volume of machined metal beads held by each braincase. We then calculated the correlation between this volume estimate and a proxy for cross-sectional area (i.e. area of an ellipse, estimated as CSA = (BD/2)*(BB/2)*π) to assess the interpretability of our BD analyses. To reduce potential biases toward seasonal differences and from users, all external measurements were taken by S.L. and all braincase volume estimates were made by L.K., both without *a priori* knowledge of the date of collection.

### 2.3. Statistical analyses

For our analyses, we only included data from intact specimens that met our data attribute criteria, where all three linear measurements could be made, and for which we could confidently identify their sex, age category, and location and date of collection. These requirements reduced the pool of potential specimens (988 stoats and 1466 weasels) to our final dataset containing 512 and 847 stoat and weasel skull specimens, respectively, and we draw our conclusions from these data (Dryad: http://dx.doi.org/10.5061/dryad.g57g1 [40]).

We first explored differences between species, sexes and age groups in each skull metric. Because these data were non-normally distributed, we used a bootstrapping procedure (electronic supplementary material, code S1) (*boot* package, v. 1.3-18; [45], within program R, v. 3.3.1; [46]) to investigate whether the presence or lack of significant differences between groups is an artefact of the data distribution. We produced a new distribution of possible skull metric measurements sampled with replacement from the observed data, producing a normally distributed, new sample of equal size to the observed for each group of interest; e.g. a new distribution of BD for juvenile female weasels. We then performed a *t*-test between each group of interest using the bootstrapped samples. We repeated the sampling and *t*-test procedure for each group comparison for 5000 iterations, tallying the number of times when the bootstrapped procedure produced *t*-values greater than or equal to the observed *t*-value (absolute value). The tally divided by 5000 is our *p*-value. Significant *p*-values (less than 0.05) indicate ‘true’ differences in observed means that are not artefacts of the observed data's non-normal distribution.

We used GAMs [42,47] to quantify size and shape patterns in CBL, BD and BD_s_ and to identify the drivers affecting these patterns. GAMs are semi-parametric generalizations of linear regression models that allow for both linear and nonlinear relationships between the response and predictors and include a penalizing smoothing function that allows for nonlinearity in the relationships [42]. We applied a smoothing term on the day of the year when the specimen was collected with a cyclic cubic regression spline (to account for the cyclical nature of calendar date) with four knots [42]. We elected not to increase the number of knots in an attempt to force the model to predict a single peak within the adults, as predicted by Dehnel's phenomenon, and limited the number of effective degrees of freedom to a maximum of 2 to reduce the potential for model over-fitting [42]. We first ran the full model that included all predictors (i.e. age, origin, sex and their interactions) and the day of the year (with a smoothing term) and then used the second-order Akaike's Information Criterion (AICc; [48,49]) to select the combination of variables and their interactions that best explained the observed patterns. We ran these models for each species separately. We performed an ANOVA on the optimal model to determine the relative influence of each predictor on the shape of the response. We then re-ran the optimal model, but with the addition of an interaction between the smoothing term and each factor separately and compared the AICc values of these models to those of the same model except without the interaction. Instances where the inclusion of the smoothing term–factor interaction improved the model performance would suggest that the response is better described for each level of the factor (e.g. for females and males) separately, rather than combined. Lastly, we inspected the residual diagnostics of the optimal models to assess the normality and homogeneity of the residuals and the model fit. All analyses were conducted within program R (v. 3.3.1; [46]) using the *mgcv* (v. 1.8-6; [50]) and *MuMIn* (v. 1.15.6; [51]) packages for executing and comparing the GAMs.

## 3. Results and discussion

### 3.1. Braincase volume

Braincase volume correlates strongly with both BD_s_ and with CSA_s_ in both species (stoat BD_s_: Spearman's *ρ* = 0.792, *p* < 0.001; stoat CSA_s_: Spearman's *ρ* = 0.937, *p* < 0.001; weasel BD_s_: Spearman's *ρ* = 0.709, *p* < 0.001; weasel CSA_s_: Spearman's *ρ* = 0.935, *p* < 0.001) (electronic supplementary material, figure S2), supporting the inferential utility of our linear measures. Therefore, we present our data using linear metrics that are directly comparable to the data presented by Dehnel [17] and others (e.g. [18–21]). Linear metrics are also important for logistical purposes; making a reasonable estimate for braincase volume is very challenging because the braincase must be both completely intact and free of tissue and debris. These requirements are difficult to fulfil for even the best-maintained collections; nonetheless, we were able to measure braincase volume on 36% and 42% of the weasel and stoat specimens, respectively, that we included in our analyses from the Muséum d‘Histoire Naturelle in Brussels, Belgium.

### 3.2. Patterns in *Mustela* skull length

**Figure 1.**
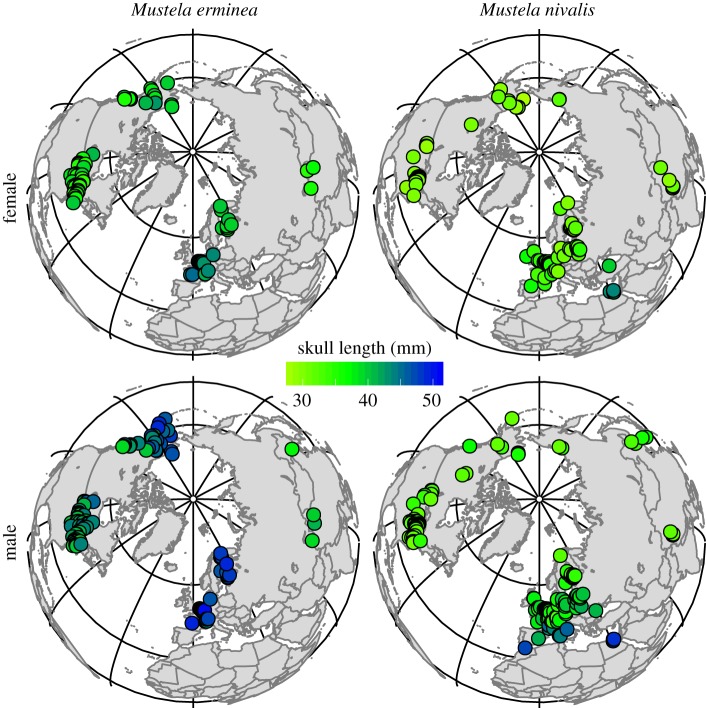
Adult *M. erminea* (*n* = 323) and *M. nivalis* (*n* = 488) specimen collection locations. Circle fill colour indicates shortest to longest condylobasal lengths (yellow to blue, respectively), showing sexual size dimorphism between females and the larger males in both species and the geographical distribution of skull length across both species.

Age, latitude and sex affect variation in stoat and weasel skull lengths (table 1 and figure 1; electronic supplementary material, figure S3). Male stoats have the longest skulls, followed by female stoats, male weasels and, the smallest, female weasels (table 1) (see also [38]). The age classes were generally similar in skull length, except juvenile weasels were significantly smaller than sub-adults and adults, for both sexes (table 1; electronic supplementary material, table S3). This is not unexpected, because weasels can produce a second litter in autumn in years with high prey abundance [38], thus these differences could also be produced by including specimens that represent juveniles born in the second cohort, who probably experience restricted growth as seen in rodent species (e.g. *Microtus oeconomus*; [32]).

**Table 1.** Both *M. erminea* and *M. nivalis* show numerous sex and age-based differences in condylobasal length (mm), braincase breadth (mm) and braincase depth (mm). Juvenile braincase depths of both species and sexes are significantly greater than those of sub-adults and adults. Cases where groups are not significantly different are indicated with matching superscripts, as most (29/36) are significant.

		condylobasal length		braincase breadth		braincase depth	
	*n*	mean (±s.e.m.)	variance	mean (±s.e.m.)	variance	mean (±s.e.m.)	variance
*M. erminea*	512	41.68 (0.17)	15.53	19.56 (0.09)	3.784	13.75 (0.08)	3.042
female	227	38.81 (0.21)	9.745	18.56 (0.11)	2.963	12.97 (0.11)	2.722
juvenile	44	38.00 (0.34)^a^	5.068	19.90 (0.22)	2.207	14.32 (0.14)	0.920
sub-adult	55	38.63 (0.46)^a^	11.74	18.78 (0.23)	2.890	13.54 (0.20)	2.263
adult	128	39.16 (0.28)^a^	10.27	18.00 (0.14)	2.235	12.26 (0.13)	2.295
male	285	43.97 (0.17)	8.357	20.35 (0.10)	3.030	14.37 (0.09)	2.429
juvenile	29	43.76 (0.42)^b^	5.199	22.11 (0.26)	1.888	15.87 (0.17)	0.834
sub-adult	61	43.65 (0.37)^b^	8.578	20.92 (0.21)	2.733	15.22 (0.18)	1.893
adult	195	44.10 (0.21)^b^	8.776	19.91 (0.11)	2.553	13.89 (0.10)	2.050
*M. nivalis*	847	34.24 (0.13)	13.87	15.95 (0.05)	2.377	11.12 (0.04)	1.469
female	351	31.65 (0.12)	5.419	14.99 (0.06)	1.239	10.36 (0.05)	0.845
juvenile	63	30.46 (0.21)	2.802	15.72 (0.10)^e^	0.579	11.17 (0.07)	0.281
sub-adult	98	31.84 (0.18)^c^	3.073	15.44 (0.09)^e^	0.815	10.68 (0.07)	0.514
adult	190	31.95 (0.19)^c^	6.961	14.51 (0.08)	1.173	9.93 (0.06)	0.752
male	496	36.07 (0.15)	11.78	16.63 (0.06)	2.062	11.66 (0.05)	1.220
juvenile	69	34.24 (0.29)	5.912	17.27 (0.13)^f^	1.139	12.21 (0.10)^g^	0.625
sub-adult	129	35.79 (0.23)^d^	6.691	17.02 (0.10)^f^	1.296	12.00 (0.07)^g^	0.664
adult	298	36.61 (0.22)^d^	14.29	16.31 (0.09)	2.354	11.38 (0.07)	1.404

Adult stoat skull lengths are positively correlated with latitude (female: Spearman's *ρ* = 0.348, *p* < 0.001; male: Spearman's *ρ* = 0.535, *p* < 0.001) as predicted by Bergmann's Rule, but unlike *Sorex* species [52]. Adult female weasel skull lengths are negatively correlated with latitude (Spearman's *ρ* = −0.155, *p* = 0.033), whereas adult male weasels are not (figure 1). A correlation is a simple statistic however, when in reality the geographical patterns in both species are complex and deserving of further investigation. For example, Erlinge [53] found that stoats in Europe were smaller at northern latitudes, whereas stoats in North America are generally larger in the north [38]. Both species exhibit substantial size variation across their ranges [38,41,54–57] and even within smaller, regional scales [26,43]. Some of this variation arises from local adaptations, including subspecies attributes or extreme cases (e.g. the urban *M. n. subpalmata* in Cairo, Egypt; figure 1), although these patterns are likely to be driven by climatic factors and resource availability [43].

### 3.3. Ontogenetic patterns in *Mustela* braincase depth

**Figure 2.**
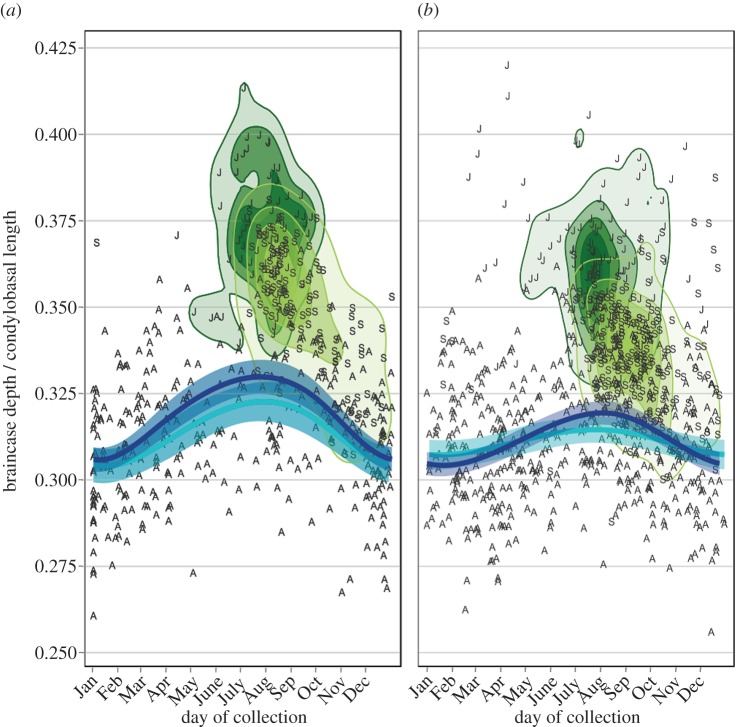
Juveniles (‘J’; dark green density contours) and sub-adults (‘S’; light green density contours) have significantly taller braincases than adults (‘A’; no density contours) in both species. GAM predictions using only adult specimens show seasonal patterns in the standardized braincase depths of both females (turquoise) and males (dark blue) of *M. erminea* (*a*) and *M. nivalis* (*b*). In both species, males tend to show greater seasonal changes than females (shading indicates 95% Bayesian confidence intervals).

Both female and male juveniles of each species exhibit a growth overshoot in both absolute (table 1) and standardized BD (figure 2; electronic supplementary material, figure S4). This overshoot varies in magnitude between the sexes and species, as juvenile mean BD for female and male stoats are 16.8% and 14.3% larger than adults, respectively, while weasels exhibit similar, but slightly less differences: 12.5% and 7.3%, respectively. This unusual growth overshoot in absolute BD is similar to what Schmidt [26] reported for weasels, but has not been reported previously for stoats. Age and its interaction with sex were retained in the four best performing GAMs for both species (table 2). Age also had the largest effect size of the parametric model terms (electronic supplementary material, table S1), where adult specimens of both species had the smallest BD_s_ (figure 2), and the three age classes each affected the shape of the response curve for BD_s_ differently (electronic supplementary material, table S2). This suggests that juveniles have both a deeper than expected braincase, given their skull length, but also that juveniles experience a growth overshoot in absolute BD that then decreases again as the individual reaches adulthood (figure 2). It is important to distinguish between ontogenetic changes in shape (i.e. relative depth, BD_s_) versus an overshoot in absolute BD. A shift in shape and proportion in growing individuals has long been recognized in mammals [3,10], including our study species [26,44,58]. Such a re-organization of the skull from one with a bulbous braincase and short rostrum to a flatter braincase and elongated rostrum has clear adaptive advantages, particularly for Mustelids which possess the highest bite forces relative to body size of all carnivores [59,60]. However, there is only a slight effect of age on CBL (table 1), but a clear age pattern in BD. This suggests that the majority of the ontogenetic shape change in these species is BD rather than skull length, including an overshoot in absolute depth of the skull and potentially the brain.

**Table 2.** The optimal GAM to predict BD_s_ in both *M. erminea* and *M. nivalis*, ranked by model weight. Explained deviance (adjusted; *R*^2^), effective degrees of freedom (edf), log-likelihood (log(L)), second-order AIC (AICc), change in AICc (ΔAICc) and model weight (w) are provided.

intercept	model terms							*R*^2^	edf	log(L)	AICc	ΔAICc	w
*M. erminea*													
0.3126	s(doy)	age	origin	sex	age*sex			0.700	13.9	1406.2	−2782	0.00	0.814
0.3109	s(doy)	age	origin	sex	age*sex		origin*sex	0.705	19.9	1410.6	−2778	3.94	0.113
0.3099	s(doy)	age	origin	sex	age*sex	age*origin		0.711	25.9	1416.7	−2776	5.16	0.062
0.3078	s(doy)	age	origin	sex	age*sex	age*origin	origin*sex	0.717	31.9	1421.7	−2773	8.63	0.011
*M. nivalis*													
0.3247	s(doy)	age	origin	sex	age*sex			0.600	12.9	2305.6	−4582	0.00	0.537
0.3256	s(doy)	age	origin	sex	age*sex	age*origin		0.608	21.9	2314.4	−4581	1.25	0.287
0.3242	s(doy)	age	origin	sex	age*sex		origin*sex	0.604	17.9	2309.2	−4579	3.28	0.104
0.3252	s(doy)	age	origin	sex	age*sex	age*origin	origin*sex	0.611	26.9	2318.0	−4578	4.66	0.052

### 3.4. Seasonal patterns in adult *Mustela* braincase depth

We identified seasonally driven, reversible changes in the BD_s_ of adults of both species (figure 2; electronic supplementary material, table S4). Including only adults in this analysis allowed us to reduce the potentially confounding effect of coincident ontogenetic patterns. These seasonal patterns suggest that adults of both species are able to modify the shape of their skulls after their sutures are fused and their skull is fully ossified. The influence of season on adult BD_s_ is significant and nonlinear (table 2); treating the day of the year as a linear factor by removing the smoothing function on the day of the year as with a generalized linear model greatly reduced the model's performance compared to the optimal GAM for each species (stoat ΔAICc = 91, weasel ΔAICc = 30; compared with a ΔAICc of 3.9 and 1.3, respectively, to the next best models that retained the smoothing term; table 2). In short, the braincase depths of both stoats and weasels decrease from their maxima as juveniles, reach their minima during their first winter, then regrow during their first summer as adults, and finally decrease again during their second winter (figure 2). This pattern is not an annual selection event that favours smaller individuals during the winter as adults with relative small BD_s_ in summer are relatively rare (figure 2). The seasonal patterns could be partially affected by individuals of different sizes having differing propensities to enter baited traps, but this would not explain the summer size peak driven by the simultaneous capture of larger individuals and the relative rarity of smaller individuals.

Both female and male adult stoats exhibited the similar and significant summer regrowth peak in BD_s_ (figure 2; electronic supplementary material, table S2), with males consistently larger than females (electronic supplementary material, table S1). Adult male weasels did exhibit a significant summer peak in BD_s_ also, but females did not (figure 2; electronic supplementary material, table S2), and the sexes did not differ in mean BD_s_ (electronic supplementary material, table S1). We believe these interspecific and intraspecific differences arise from the species' reproductive strategies. Weasels are classic examples of *r*-strategists as they are small, short-lived, mature early, inhabit variable habitats and rely on cyclical prey populations [61]. As such, female weasels will produce two cohorts in years with high prey abundance, and in these years, females born in the first cohort may even produce their own litters before their first winter [38]. Thus, female weasels invest their energy into their offspring, rather than investing energy into themselves [62]. Male weasels, however, can invest in themselves as they provide no paternal care and should strive for a larger body size in order to fend off smaller males and to suppress females during copulation. Stoats are less opportunistic strategists than weasels. Unlike weasels, female stoats cannot produce a litter within their first year and are prevented by delayed implantation from producing more than one litter per year beyond that [38]. Thus, both female and male stoats are able to invest surplus energy into their own somatic maintenance. Additionally, because their mating opportunities are more limited than for the weasels, securing opportunities becomes important for male stoats and in these situations larger males are more successful [53].

**Figure 3.**
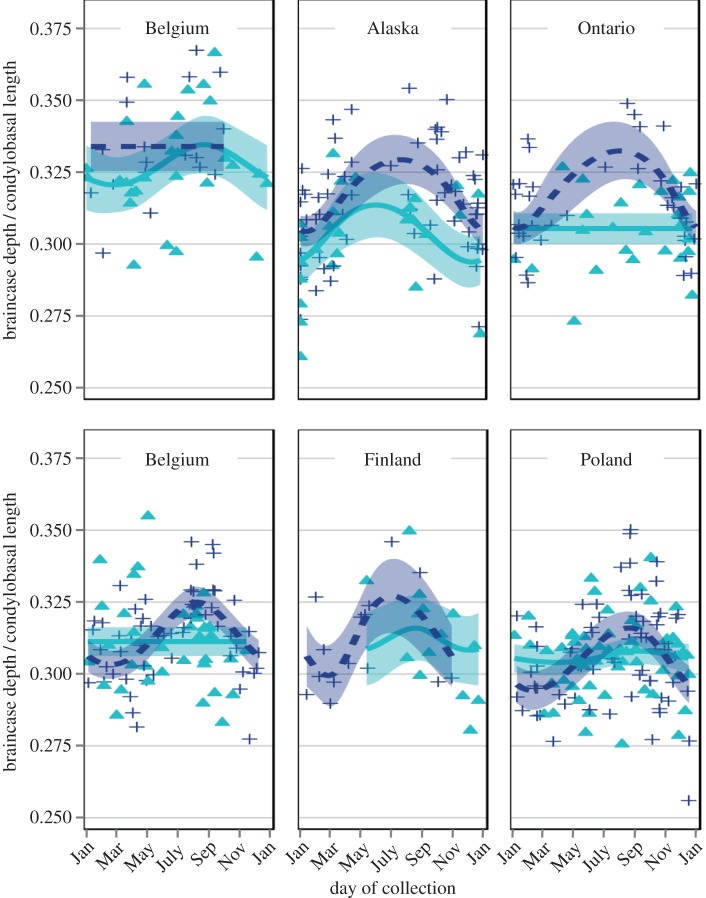
Seasonal patterns in the standardized braincase depths of adult female (turquoise triangles, line and fill) and male (dark blue plus-signs, line and fill) *M. erminea* (top row) and *M. nivalis* (bottom row) occur across the geographical range of each species, but also within several smaller geographical regions. GAM predictions suggest that the presence, magnitude and timing of the seasonal patterns varies between sexes, species and geographical regions (shading indicates 95% Bayesian confidence intervals). See electronic supplementary material, table S5 for sample sizes, *p*-values and model attributes.

We also identified geographical variation in the presence, timing and magnitude of the changes in BD_s_ of adult weasels and stoats (figure 3) in addition to the geographical and latitudinal patterns in skull length (i.e. species-level size variation). Importantly, these patterns are not only developmental effects (as seen in figure 2), as we included only adults in this analysis (electronic supplementary material, table S5), and they are not artefacts of inverse seasonal changes in CBL alone (electronic supplementary material, figure S5). The shifts in the timing of the peak and differences in the magnitude might suggest climatic modulators of the genetically fixed seasonal patterns, where populations from areas with more severe winter conditions (e.g. Alaska, Finland or Ontario) would show a more sinuous seasonal pattern, as seen in figure 3.

## 4. Conclusion

We present evidence of two rare phenomena: an absolute growth overshoot of juvenile BD and a seasonally driven BD change in the adult skull. Although postnatal changes in skull shape and size are ubiquitous in many vertebrates, species rarely exhibit a bidirectional change, particularly as adults. Examples of body size changes exist, but are unidirectional responses to severe reductions in resource availability (e.g. drought) and are unlike the seasonal pattern we observe (e.g. salamanders [63], cichlid fishes [64], salmonids [65] and tortoises [66]). El Niño events triggered body length decreases in adult marine iguanas (*Amblyrhyncus cristatus*) that later re-grew, but are an example of a reactive bidirectional size change [67]. Although bidirectional changes in bird brain volume driven by adult neurogenesis occur (e.g. [68]), similar changes within the skull have not been documented.

The seasonal patterns in BD_s_ that we observed are additional to developmental changes and, as the specimens represent a global distribution collected over the last century, are not driven by reactions to episodic or unpredictable fluctuations in resource availability. We hypothesize that the observed seasonal patterns reflect an alternative over-wintering strategy that is genetically fixed, but the magnitude of these fluctuations is affected by the severity of the conditions. To further understand the mechanisms and evolution of this strategy, future efforts should consider repeated measures on living individuals to quantify intra-individual changes, and molecular investigations to quantify the mechanisms of these changes. Lastly, our observations of Dehnel's phenomenon occurring in two carnivore species that are taxonomically distantly related to *Sorex* shrews, provides strong support for the convergent evolution of this phenomenon. Future efforts working within this convergent evolutionary framework should provide fruitful research directives for ecologists, evolutionary biologists and researchers in human medicine.

## Supplementary Material

Table S1. Summary of the optimal generalized additive model of BDs per species. For each factor, degrees of freedom (df) are indicated, but for the smooth term df indicates effective degrees of freedom. The test statistic listed is either the F-statistic for the factor level or the t-value for each level within a factor.

## Supplementary Material

Table S2. ANOVAs on the optimal models, per species, with the addition of an interaction between the smooth term (day of year) and each factor, suggest (via p< 0.05) that the predictions would be improved by applying a smooth term to each level within a factor separately, except for sex in M. erminea. Thus, as the different levels within most factors differ in the shape of their response curve, we find support to consider factor levels separately.

## Supplementary Material

Table S3. We bootstrapped t-tests (5000 iterations, with replacement) between age categories for each sex and species combination to produce a normally distributed random sample from the observed non-normally distributed measurement data and compared the likelihood (p-value) of calculating the number of possible t-values (i.e., tb; mean t-value from bootstrapped simulations) that was greater than or equal to the observed absolute t-value (i.e., to). Significant p-values (<0.05) indicate “true” differences in observed means that are not artifacts of the observed data distribution. These bootstrapped t-tests suggest braincase depth growth overshoots across sexes and species in juveniles, as these braincases are significantly taller than all other age categories, yet similar to or significantly shorter in CBL to all other age categories (see metric means in Table 1). The results presented here suggest these significant differences persist despite the non-normal distribution of the data. Bold to values indicate significant differences between groups via t-tests on observed data directly.

## Supplementary Material

Table S4. The optimal generalized additive model to predict adult BDs in both M. erminea (n = 323) and M. nivalis (n = 488), ranked by model weight. Explained deviance (adjusted; R2), effective degrees of freedom (edf), log-likelihood (log(L)), second-order AIC (AICc), change in AICc (ΔAICc), and model weight (w) are provided.

## Supplementary Material

Table S5. Optimal generalized additive models of adult BDs per species-sex-origin combination. These results are displayed graphically in Figure 3. Only the specimen's collection day of the year with a smooth term is included as an explanatory variable here. For each origin-sex combination per species (row), sample size (n), standard error (SE), intercept, explained deviance (adjusted; R2), effective degrees of freedom (edf), log-likelihood (log(L)), second-order AIC (AICc), and the significance level of the smoothed day of the year (p-value) are provided. Despite low adjusted R2 values, p-values less than 0.05 suggest significantly non-linear patterns in BDs. Note: sample sizes do not sum to the sample sizes of each species (M. erminea = 512, M. nivalis = 847) since this analysis focuses solely on a subset of the entire data set for which we have sufficient sample sizes and variation in day of year, within a sex-origin combination.

## Supplementary Material

Code S1. Program R code for executing bootstrapping of t-tests to compare skull metrics between age, sex, species, and origin groupings.

## Supplementary Material

Figure S1. Stoat and weasel specimens were assigned to one of three age classes based on (i) the degree of ossification of the nasal sutures, (ii) the roundness of the braincase, (iii) the progression of development of the sagittal and nuchal crests, and (iv) to lesser degrees tooth ware and the presence of damage from Skrjabingylus nasicola worms near the postorbital processes of the frontal bones (indicated below in C with at the arrow). Individuals with clearly visible nasal sutures (i.e., partially ossified), rounded and generally smooth braincases, whose sagittal crest was not present and whose nuchal crests are faint were assigned as juveniles (A). Subadults (B) were characterized by mostly fused and ossified nasal sutures, a less rounded and smooth braincase posterior, whose nuchal crests were developing, and possibly display early stages of S. nasicola worm damage. Specimens were deemed adults (C) if the nasal sutures were fully fused, the posterior of their braincase was irregularly shaped and less rounded with developed nuchal crests as well as the sagittal crest in males, and often contained damage from the S. nasicola worms. The damage from these worms often prevented us from using a more quantitative method of aging, for example the ratio of the interorbital width to the postorbital width. Data from these skulls can be found in the supplementary dataset with this manuscript, using catalogue numbers 38738 (A), 161084 (B), and 51148 (C) and are maintained in the collections of the Mammal Research Institute.

## Supplementary Material

Figure S2. Standardized braincase volume was positively correlated with braincase cross-sectional area (top) and braincase depth (bottom) in M. erminea (blue; Spearman's rho = 0.937 and 0.792, p < 0.001, respectively) and M. nivalis (orange; Spearman's rho = 0.935 and 0.709, p < 0.001, respectively) specimens. Standard error indicated with shading. See Methods for braincase volume and area calculations.

## Supplementary Material

Figure S3. Frequency plots showing the distributions of each skull metric (condylobasal length, left; braincase breadth, center; and braincase depth, right) per species (M. erminea, top; M. nivalis, bottom) for each age and sex combination. Vertical dashed lines indicate means. Note: scales are consistent between species within metrics, but differ between metrics.

## Supplementary Material

Figure S4. Boxplots showing the distribution and mean of braincase depth (top) and standardized braincase depth (BDs, bottom) of M. erminea (left) and M. nivalis (right) per age, sex, and origin grouping. Alternating grey vertical bars distinguish origins. Sexes (F, female; M, male) are separated by color, with increasing darkness within sex and origin indicating increasing age category (J, juvenile; S, subadult; A, adult). Outliers indicated with increased point outline thickness. There are no juvenile male M. erminea from Ontario included within our analyses. Note: scales vary between species and metrics.

## Supplementary Material

Figure S5. Generalized additive model predictions for seasonal patterns in the condylobasal lengths (left column), braincase depths (center column), and standardized braincase depths (right column) for adult females (turquoise and triangles) and males (dark blue and plus-signs) of both M. erminea (top row) and M. nivalis (bottom row). Here we show that the seasonal pattern in standardized braincase depth is not driven by disproportionate nor independent changes in condylobasal length or braincase depth alone. Note: species are drawn on same y-axis per size metric, but the scales differ between the metrics.

## Data accessibility

The dataset supporting this article has been deposited at the Dryad Digital Repository: http://dx.doi.org/10.5061/dryad.g57g1 [40].
