# Supplementary material for: Growth overshoot and seasonal size changes in the skulls of two weasel species
Source: R Soc Open Sci. 2017 Jan 25;4(1):160947. doi: 10.1098/rsos.160947 (PMC5319358; doi:10.1098/rsos.160947)
Supplement: Table S1. Summary of the optimal generalized additive model of BDs per species. For each factor, degrees of freedom (df) are indicated, but for the smooth term df indicates effective degrees of freedom. The test statistic listed is either the F-statistic for the factor level or the t-value for each  [file rsos160947supp1.docx]

**Table S1.** Summary of the optimal generalized additive model of BD_s_ per species. For each factor, degrees of freedom (df) are indicated, but for the smooth term df indicates effective degrees of freedom. The test statistic listed is either the F-statistic for the factor level or the t-value for each level within a factor.

| Factor (level) | estimate | SEe-02 | df | test statistic | *p* |
| --- | --- | --- | --- | --- | --- |
| *M. erminea* |  |  |  |  |  |
| intercept | 0.313 | 0.188 |  | 165.97 | < 0.001 |
| sex |  |  | 1 | 10.08 | 0.002 |
| male | 0.006 | 0.183 |  | 3.18 | 0.002 |
| age |  |  | 2 | 172.81 | < 0.001 |
| juvenile | 0.053 | 0.301 |  | 17.64 | < 0.001 |
| subadult | 0.030 | 0.274 |  | 11.09 | < 0.001 |
| origin |  |  | 6 | 7.58 | < 0.001 |
| Belgium | 0.011 | 0.223 |  | 4.73 | < 0.001 |
| Finland | -0.002 | 0.298 |  | -0.61 | 0.540 |
| Michigan | -0.001 | 0.288 |  | -0.39 | 0.698 |
| New York | -0.005 | 0.258 |  | -2.06 | 0.040 |
| Ontario | < -0.001 | 0.208 |  | -0.04 | 0.969 |
| other | 0.008 | 0.298 |  | 2.54 | 0.011 |
| age*sex |  |  | 2 | 11.51 | < 0.001 |
| male*juvenile | -0.020 | 0.419 |  | -4.66 | < 0.001 |
| male*subadult | -0.008 | 0.346 |  | -2.18 | 0.030 |
| s(day of year) |  |  | 1.94 | 49.45 | < 0.001 |
| *M. nivalis* |  |  |  |  |  |
| intercept | 0.325 | 0.282 |  | 115.30 | < 0.001 |
| sex |  |  | 1 | 0.06 | 0.803 |
| male | < 0.001 | 0.150 |  | 0.25 | 0.803 |
| age |  |  | 2 | 261.33 | < 0.001 |
| juvenile | 0.054 | 0.240 |  | 22.42 | < 0.001 |
| subadult | 0.024 | 0.210 |  | 11.47 | < 0.001 |
| origin |  |  | 5 | 15.77 | < 0.001 |
| Belgium | -0.014 | 0.293 |  | -4.62 | < 0.001 |
| Finland | -0.112 | 0.351 |  | -3.30 | < 0.001 |
| Michigan | -0.002 | 0.359 |  | -0.50 | 0.619 |
| Poland | -0.019 | 0.291 |  | -6.44 | < 0.001 |
| other | -0.011 | 0.296 |  | -3.92 | < 0.001 |
| age*sex |  |  | 2 | 6.90 | 0.001 |
| male*juvenile | -0.011 | 0.318 |  | -3.59 | < 0.001 |
| male*subadult | < -0.001 | 0.263 |  | -0.05 | 0.960 |
| s(day of year) |  |  | 1.90 | 16.70 | < 0.001 |
