# Supplementary material for: Growth overshoot and seasonal size changes in the skulls of two weasel species
Source: R Soc Open Sci. 2017 Jan 25;4(1):160947. doi: 10.1098/rsos.160947 (PMC5319358; doi:10.1098/rsos.160947)
Supplement: Table S2. ANOVAs on the optimal models, per species, with the addition of an interaction between the smooth term (day of year) and each factor, suggest (via p< 0.05) that the predictions would be improved by applying a smooth term to each level within a factor separately, except for sex in M. ermine [file rsos160947supp2.docx]

**Table S2.** ANOVAs on the optimal models, per species, with the addition of an interaction between the smooth term (day of year) and each factor, suggest (via *p* < 0.05) that the predictions would be improved by applying a smooth term to each level within a factor separately, except for sex in *M. erminea*. Thus, as the different levels within most factors differ in the shape of their response curve, we find support to consider factor levels separately.

| Factor:level | | edf | F | *p* |
| --- | --- | --- | --- | --- |
| *M. erminea* | | | | |
|  | s() * age | 1.878 | 35.74 | < 0.001 |
|  | juvenile | < 0.001 | 0.0 | 1.000 |
|  | subadult | 1.101 | 2.33 | 0.018 |
|  | adult | < 0.001 | 0.0 | 0.251 |
|  | s() * origin | 1.704 | 19.76 | < 0.001 |
|  | Alaska | 1.440 | 3.332 | 0.007 |
|  | Belgium | < 0.001 | 0.0 | 0.946 |
|  | Finland | < 0.001 | 0.0 | 0.355 |
|  | Michigan | 1.040 | 2.018 | 0.020 |
|  | New York | < 0.001 | 0.0 | 0.557 |
|  | Ontario | 0.717 | 0.673 | 0.135 |
|  | other | < 0.001 | 0.0 | 0.711 |
|  | s() * sex | 1.934 | 49.3 | < 0.001 |
|  | female | < 0.001 | 0.0 | 0.650 |
|  | male | < 0.001 | 0.0 | 0.312 |
| *M. nivalis* | | | | |
|  | s() * age | < 0.001 | 0.0 | 0.218 |
|  | juvenile | 1.016 | 1.418 | 0.063 |
|  | subadult | 1.608 | 5.975 | < 0.001 |
|  | adult | 1.903 | 18.76 | < 0.001 |
|  | s() * origin | 1.277 | 2.554 | 0.010 |
|  | Alaska | 1.852 | 10.44 | < 0.001 |
|  | Belgium | 0.432 | 0.261 | 0.258 |
|  | Finland | 1.646 | 4.878 | 0.002 |
|  | Michigan | < 0.001 | 0.0 | 0.769 |
|  | Poland | 1.600 | 4.395 | 0.002 |
|  | other | < 0.001 | 0.0 | 0.934 |
|  | s() * sex | 1.433 | 2.778 | 0.016 |
|  | female | < 0.001 | 0.0 | 0.093 |
|  | male | 1.780 | 6.944 | < 0.001 |
