# Supplementary material for: Growth overshoot and seasonal size changes in the skulls of two weasel species
Source: R Soc Open Sci. 2017 Jan 25;4(1):160947. doi: 10.1098/rsos.160947 (PMC5319358; doi:10.1098/rsos.160947)
Supplement: Table S3. We bootstrapped t-tests (5000 iterations, with replacement) between age categories for each sex and species combination to produce a normally distributed random sample from the observed non-normally distributed measurement data and compared the likelihood (p-value) of calculating the numbe [file rsos160947supp3.docx]

|  |  | |  | |  | *Mustela erminea* | | | | | | *Mustela nivalis* | | | | | |  |
| --- | --- | --- | --- | --- | --- | --- | --- | --- | --- | --- | --- | --- | --- | --- | --- | --- | --- | --- |
|  | Age comparisons | | | | | female | | | male | | | female | | | male | | |  |
|  |  |  | |  | | t_o_ | t_b_ | *p* | t_o_ | t_b_ | *p* | t_o_ | t_b_ | *p* | t_o_ | t_b_ | *p* | |
| CBL | juvenile | : | | subadult | | 1.093 | 1.098 | 0.141 | 0.203 | 0.193 | 0.417 | **4.991** | 4.950 | <0.001 | **4.192** | 4.186 | <0.001 | |
|  | subadult | : | | adult | | 0.988 | 0.978 | 0.166 | 1.036 | 1.021 | 0.156 | 0.434 | 0.408 | 0.329 | 2.594 | 2.576 | 0.008 | |
|  | juvenile | : | | adult | | 2.628 | 2.613 | 0.007 | 0.701 | 0.703 | 0.245 | **5.223** | 5.132 | <0.001 | **6.495** | 6.428 | <0.001 | |
| Breadth | juvenile | : | | subadult | | **3.492** | 3.487 | <0.001 | **3.602** | 3.578 | 0.001 | 2.065 | 2.075 | 0.020 | 1.548 | 1.570 | 0.057 | |
|  | subadult | : | | adult | | **2.941** | 2.974 | 0.003 | **4.212** | 4.217 | <0.001 | **7.755** | 7.765 | <0.001 | **5.284** | 5.294 | <0.001 | |
|  | juvenile | : | | adult | | **7.270** | 7.278 | <0.001 | **7.894** | 7.909 | <0.001 | **9.735** | 9.793 | <0.001 | **6.146** | 6.181 | <0.001 | |
| Depth | juvenile | : | | subadult | | **3.122** | 3.120 | 0.002 | **2.670** | 2.655 | 0.007 | **5.000** | 4.999 | <0.001 | 1.737 | 1.731 | 0.040 | |
|  | subadult | : | | adult | | **5.258** | 5.282 | <0.001 | **6.540** | 6.557 | <0.001 | **7.779** | 7.676 | <0.001 | **6.289** | 6.278 | <0.001 | |
|  | juvenile | : | | adult | | **10.43** | 10.417 | <0.001 | **10.02** | 10.015 | <0.001 | **13.504** | 13.513 | <0.001 | **7.085** | 7.109 | <0.001 | |

**Table S3.** We bootstrapped t-tests (5000 iterations, with replacement) between age categories for each sex and species combination to produce a normally distributed random sample from the observed non-normally distributed measurement data and compared the likelihood (*p*-value) of calculating the number of possible t-values (i.e., t_b_; mean t-value from bootstrapped simulations) that was greater than or equal to the observed absolute t-value (i.e., t_o_). Significant *p*-values (<0.05) indicate “true” differences in observed means that are not artifacts of the observed data distribution. These bootstrapped t-tests suggest braincase depth growth overshoots across sexes and species in juveniles, as these braincases are significantly taller than all other age categories, yet similar to or significantly shorter in CBL to all other age categories (see metric means in table 1). The results presented here suggest these significant differences persist despite the non-normal distribution of the data. Bold t_o_ values indicate significant differences between groups via t-tests on observed data directly.
