# Supplementary material for: Growth overshoot and seasonal size changes in the skulls of two weasel species
Source: R Soc Open Sci. 2017 Jan 25;4(1):160947. doi: 10.1098/rsos.160947 (PMC5319358; doi:10.1098/rsos.160947)
Supplement: Table S4. The optimal generalized additive model to predict adult BDs in both M. erminea (n = 323) and M. nivalis (n = 488), ranked by model weight. Explained deviance (adjusted; R2), effective degrees of freedom (edf), log-likelihood (log(L)), second-order AIC (AICc), change in AICc (ΔAICc), and mo [file rsos160947supp4.docx]

**Table S4.** The optimal generalized additive model to predict adult BD_s_ in both *M. erminea* (n = 323) and *M. nivalis* (n = 488), ranked by model weight. Explained deviance (adjusted; R^2^), effective degrees of freedom (edf), log-likelihood (log(L)), second-order AIC (AICc), change in AICc (ΔAICc), and model weight (w) are provided.

| intercept | model terms | | | | R^2^ | edf | log(L) | AICc | ΔAICc | w |
| --- | --- | --- | --- | --- | --- | --- | --- | --- | --- | --- |
| *M. erminea* |  |  |  |  |  |  |  |  |  |  |
| 0.3022 | s(doy) | origin | sex | origin*sex | 0.285 | 15.9 | 877.9 | -1720 | 0.00 | 0.502 |
| 0.3062 | s(doy) | origin | sex |  | 0.269 | 9.88 | 871.3 | -1720 | 0.04 | 0.491 |
| 0.3106 | s(doy) | origin |  |  | 0.247 | 8.87 | 865.9 | -1711 | 8.72 | 0.006 |
| *M. nivalis* |  |  |  |  |  |  |  |  |  |  |
| 0.3246 | s(doy) | origin |  |  | 0.140 | 7.88 | 1320.5 | -2623 | 0.00 | 0.725 |
| 0.3243 | s(doy) | origin | sex |  | 0.138 | 8.88 | 1320.6 | -2621 | 1.96 | 0.272 |
| 0.3242 | s(doy) | origin | sex | origin*sex | 0.131 | 13.9 | 1321.2 | -2612 | 11.3 | 0.003 |
