# Supplementary material for: Growth overshoot and seasonal size changes in the skulls of two weasel species
Source: R Soc Open Sci. 2017 Jan 25;4(1):160947. doi: 10.1098/rsos.160947 (PMC5319358; doi:10.1098/rsos.160947)
Supplement: Table S5. Optimal generalized additive models of adult BDs per species-sex-origin combination. These results are displayed graphically in Figure 3. Only the specimen's collection day of the year with a smooth term is included as an explanatory variable here. For each origin-sex combination per speci [file rsos160947supp5.docx]

**Table S5.** Optimal generalized additive models of adult BD_s_ per species-sex-origin combination. These results are displayed graphically in Figure 3. Only the specimen’s collection day of the year with a smooth term is included as an explanatory variable here. For each origin-sex combination per species (row), sample size (n), standard error (SE), intercept, explained deviance (adjusted; R^2^), effective degrees of freedom (edf), log-likelihood (log(L)), second-order AIC (AICc), and the significance level of the smoothed day of the year (*p*) are provided. Despite low adjusted R^2^ values, p-values less than 0.05 suggest significantly non-linear patterns in BD_s_. Note: sample sizes do not sum to the sample sizes of each species (*M. erminea* = 512, *M. nivalis* = 847) since this analysis focuses solely on a subset of the entire data set for which we have sufficient sample sizes and variation in day of year, within a sex-origin combination.

| origin | sex | n | SE | intercept | R^2^ | edf | log(L) | AICc | *p* |
| --- | --- | --- | --- | --- | --- | --- | --- | --- | --- |
| *M. erminea* |  |  |  |  |  |  |  |  |  |
| Alaska | female | 27 | 0.003 | 0.302 | 0.001 | 1.60 | 75.1 | -141.6 | <0.001 |
| Alaska | male | 69 | 0.002 | 0.313 | 0.001 | 1.84 | 188.4 | -368.5 | <0.001 |
| Belgium | female | 28 | 0.003 | 0.327 | 0.001 | 1.24 | 74.6 | -141.6 | 0.075 |
| Belgium | male | 18 | 0.004 | 0.334 | 0.000 | 0.00 | 46.7 | -88.7 | 0.632 |
| Ontario | female | 26 | 0.003 | 0.305 | 0.000 | 0.00 | 75.0 | -145.5 | 0.782 |
| Ontario | male | 38 | 0.002 | 0.314 | 0.002 | 1.81 | 112.1 | -215.5 | <0.001 |
| *M. nivalis* |  |  |  |  |  |  |  |  |  |
| Belgium | female | 37 | 0.003 | 0.311 | 0.000 | 1.00 | 101.9 | -199.6 | 0.365 |
| Belgium | male | 57 | 0.002 | 0.313 | 0.001 | 1.80 | 172.9 | -337.4 | <0.001 |
| Finland | female | 14 | 0.005 | 0.312 | 0.129 | 0.61 | 37.5 | -68.1 | 0.182 |
| Finland | male | 15 | 0.003 | 0.311 | 0.002 | 1.67 | 44.9 | -79.1 | 0.024 |
| Poland | female | 61 | 0.002 | 0.306 | 0.039 | 0.93 | 179.9 | -353.6 | 0.199 |
| Poland | male | 70 | 0.002 | 0.306 | 0.001 | 1.80 | 192.1 | -376.0 | <0.001 |
